# ﻿Morphological and phylogenetic analyses reveal a new genus and two new species of Hymenochaetales (Basidiomycota) from southeast China

**DOI:** 10.3897/mycokeys.127.171179

**Published:** 2026-01-06

**Authors:** Qiu-Yue Zhang, Jin-Hua Huang, Jian-Ling Ren, Li-Hua Zhu, Lin Huang

**Affiliations:** 1 Co-Innovation Center for Sustainable Forestry in Southern China, College of Forestry, Nanjing Forestry University, Nanjing, Jiangsu 210037, China Nanjing Forestry University Nanjing China; 2 Fuijian Yangkou State-Owned Forest Farm, Nanping 353299, China Fuijian Yangkou State-Owned Forest Farm Nanping China

**Keywords:** Hymenochaetales, macrofungi, new taxa, phylogenetic analyses, taxonomy

## Abstract

The Hymenochaetales is an order in which most species are wood-inhabiting fungi, which has high phylogenetic complexity and morphological diversity, and comprises mostly polypores, corticioid, and hydnoid fungi, with some agaricoid and clavarioid fungi. During an investigation of wood-inhabiting fungi in Fujian Province, China, four corticioid fungal specimens assigned to Hymenochaetales were collected. Based on morphological characteristics and molecular evidence, a new genus *Spongoides*, and a new species, *Peniophorella subalbohymenia*, were proposed. The new genus was established to accommodate a single species *Spongoides fissurata*, characterized by its resupinate, effused, spongy basidiomata with two types cystidia, and ellipsoid basidiospores growing on living *Chamaecyparis formosensis*. The new species, *Peniophorella subalbohymenia*, is characterized by its membranaceous, white basidiomata with a smooth hymenial surface, the presence of three variable cystidia, and ellipsoid basidiospores. Illustrated descriptions of both novel taxa are provided. This study advances the understanding of Hymenochaetales diversity in China and supplements the taxonomic framework for wood-inhabiting fungi.

## ﻿Introduction

Hymenochaetales Oberw. is one of the fungal orders mainly composed of wood-inhabiting macrofungi within the class Agaricomycetes, Basidiomycota, that play crucial ecological roles in forest ecosystems (Dai 2010; Dai et al. 2021; Wu et al. 2022; Yuan et al. 2023; Zhao et al. 2024; Dai et al. 2025). The order Hymenochaetales exhibits significant variations in morphological characteristics, primarily comprising hydnoid, corticioid, and poroid (Larsson et al. 2006; Dai 2010; Korotkin et al. 2018; Zhou et al. 2022; Zhou et al. 2023; Liu et al. 2025; Zhao et al. 2025). The majority of species in the order Hymenochaetales are wood-decaying fungi that cause white rot, as well as some important plant pathogens, symbiotic fungi, and medicinal species (Wu et al. 2022; Wang and Zhou 2023; Zhao et al. 2023, 2024). Consequently, Hymenochaetales fungi represent significant biological resources, holding importance for forest ecosystem function and economic development.

Currently, more than 1,400 species have been placed in Hymenochaetales, with ongoing discoveries highlighting substantial unexplored diversity, particularly in biodiverse regions such as East Asia (Zhou et al. 2018; Wu et al. 2022; Wang et al. 2023; Zhao et al. 2024). While the species diversity has been well explored all over the world, the systematics of Hymenochaetales at the family/genus level were poorly established. At the family level, the classification of Hymenochaetales has been subject to ongoing revisions, particularly over the past fifteen years, and a total of 14 family names have been successively applied. But at the genus level, about 19 genera have never been placed in any family in Hymenochaetales (Wu et al. 2022; Wang et al. 2023; Wang and Zhou 2023).

A notable component of this order is its considerable diversity of corticioid fungi, a polyphyletic morphological group (Liu et al. 2025; Zhao et al. 2025). Historically, many corticioid genera were classified based on morphology, leading to artificial groupings. The molecular phylogenetic studies have greatly clarified the systematics of these corticioid taxa (Wu et al. 2022; Yuan et al. 2023; Zhou et al. 2023). For instance, several newly proposed families, including Hyphodontiaceae, Peniophorellaceae L.W. Zhou et al., and Schizoporaceae Jülich, primarily encompass corticioid genera, highlighting the considerable phylogenetic diversification that has occurred within this morphological group (Wang et al. 2021, 2023). Therefore, integrated morphological and phylogenetic approaches remain crucial for accurately delineating taxa among these often cryptic fungi.

The family Peniophorellaceae was established within Hymenochaetales by Wang et al. (2023), to include the genus *Peniophorella* P. Karst, typed with *P. pubera* (Fr.) P. Karst. (Karst 1889), characterized by resupinate basidiomata with ceraceous to corneus consistency, smooth to tuberculate, and odontioid hymenophore with white to brownish-yellow hymenial surface, monomitic hyphae with generative hyphae bearing clamp connections (Guan et al. 2020). It is a cosmopolitan fungal genus with 39 species level names in Index Fungorum (http://www.indexfungorum.org, accessed on 1 July 2025). With the development of molecular systematics, the positioning of *Peniophorella* in Hymenochaetales has been further strengthened. Through molecular phylogeny of *Hyphoderma* Wallr., Larsson (2007) reinstated 19 new combinations within *Peniophorella*, which phylogenetically nested within the hymenochaetoid clade, confirming its placement outside traditional classifications. Subsequent studies by Miettinen and Larsson (2011) and Telleria et al. (2012) further resolved that *Peniophorella* species formed a distinct monophyletic group, clearly separated from *Hyphoderma* sensu stricto. Justo et al. (2017) revised the family-level classification of *Peniophorella*, which classified under Hyphodermataceae in the order Hymenochaetales, solidifying its taxonomic realignment. Recently, building on these phylogenetic frameworks, Guan et al. (2020) and Deng et al. (2025) described four new species of *Peniophorella* from southwestern China using integrated morphological and molecular analyses.

An examination of four corticioid specimens collected in Fujian, China, ​confirmed that all represent undescribed species within Hymenochaetales. To identify these two specimens at a species level and determine their taxonomic position at higher ranks, careful morphological examinations and phylogenetic analyses were performed. Ultimately, this study described a new *Peniophorella* species and a new genus within incertae sedis of Hymenochaetales.

## ﻿Materials and methods

### ﻿Morphological studies

The specimens were collected from Lai Zhou Forest Farm Experimental Center, Fujian Province in South China. The obtained specimens were stored in the Forest Pathology Laboratory at Nanjing Forestry University (**NJFC**), Nanjing, China. Macromorphological characteristics were investigated based on field notes and color photos of basidiomata. Color codes were verified as proposed by Petersen (1996). Microscopy studies were conducted as described by Wang and Zhou (2023) and Deng et al. (2025). Morphological observations of reproductive structures were conducted using the Zeiss fluorescence microscope. The Nikon Digital Sight DS-L3 camera was used to photograph microscopic structures. Drawings were made with the aid of a drawing tube. Microscopic features, measurements and drawings were made from slide preparations stained with Cotton Blue and Melzer’s Reagent. Basidiospores were measured from sections cut from the hymenophore. To present the variation of basidiospores size, 5% of measurements were excluded from each end of the range and are given in parentheses. The following abbreviations are used: **IKI** = Melzer’s Reagent, **IKI−** = neither amyloid nor dextrinoid, **KOH** = 5% potassium hydroxide, **CB** = Cotton Blue, **CB−** = acyanophilous, **L** = arithmetic average of all basidiospores length, **W** = arithmetic average of all basidiospores width, **Q** = variation in the **L/W** ratios between the specimens studied, **(n = x/y)** = the number of basidiospores (x) measured from a given number of specimens (y).

### ﻿DNA extraction and sequencing

A cetyl trimethylammonium bromide (CTAB) rapid plant genome extraction kit (Aidlab Biotechnologies, Co., Ltd., Beijing, China) was used to extract DNA (Wu et al. 2020). The ITS region (nuclear ribosomal internal transcribed spacer, ITS), nuclear ribosomal large subunit (nrLSU), nuclear ribosomal small subunit (nrSSU), mitochondrial small subunit (mtSSU)) regions were amplified using the selected primer pairs: ITS5/ITS4 (White et al. 1990), LR0R/LR5 (Vilgalys and Hester 1990), NS1/NS41 (Hibbett 1996), MS1/MS2 (White et al. 1990), respectively. The PCR cycling schedule for ITS and mtSSU included an initial denaturation at 95 °C for 3 min or 94 °C for 2 min, followed by 35 cycles at 94 °C for 40 s, 54 °C for 45 s and 72 °C for 1 min, and a final extension of 72 °C for 10 min. The PCR cycling schedule for nrLSU and nrSSU included an initial denaturation at 94 °C for 1 min, followed by 35 cycles at 94 °C for 30 s, 50 °C (nrLSU), 53 °C (nrSSU) for 1 min and 72 °C for 1.5 min, and a final extension of 72 °C for 10 min (Wang et al. 2023; Zhou et al. 2023; Ma et al. 2025). All newly generated sequences in this study were deposited in GenBank and are listed in bold in Table 1.

### ﻿Phylogenetic analyses

New sequences, deposited in GenBank (http://www.ncbi.nlm.nih.gov/genbank/) (Table 1), were aligned with additional sequences retrieved from GenBank (Table 1) using BioEdit 7.0.5.3 (Hall 1999) and MAFFT v.74 (http://mafft.cbrc.jp/alignment/server/, Katoh et al. 2017). Sequences of Hymenochaetales were adopted mainly from ITS + nrLSU + nrSSU + mtSSU + rpb2 + tef1α tree topologies established by Wang and Zhou (2023) and Ma et al. (2025). Sequences of *Peniophorella* were adopted mainly from ITS + nrLSU tree topologies established by Deng et al. (2025).

**Table 1.** Sources of specimens and GenBank accession numbers for the sequences used in this study. Newly generated sequences are in bold.

Order/Family	Species	Voucher	ITS	nrLSU	nrSSU	mtSSU
**Hymenochaetales** / Chaetoporellaceae	*Echinoporia hydnophora*	LWZ 20150802-9	ON063639	ON063838	ON063768	ON063707
	*Kneiffiella eucalypticola*	LWZ 20180509-11 (Type)	NR_182823	NG_153905	—	MT326421
	*Kneiffiella subglobosa*	LWZ 20180416-6	MT319413	MT319145	—	MT326422
— / Hirschioporaceae	*Pallidohirschioporus versicolor*	Dai 19331	OQ453386	OQ474951	OQ453261	—
	*Pallidohirschioporus versicolor*	Dai 19332	OQ453387	OQ474952	OQ453262	OQ534102
	*Pallidohirschioporus versicolor*	Dai 19336	OQ453388	—	OQ453263	OQ534103
— / Hymenochaetaceae	*Basidioradulum mayi*	LWZ 20180510-18	MN017785	MN017792	ON427363	ON463756
	*Basidioradulum radula*	LWZ 20201017-62	ON063684	ON063884	ON063814	ON063747
	*Coltricia abieticola*	Cui 10321	KX364785	KX364804	KY693761	KY693823
	*Fomitiporia rhamnoides*	LWZ 20180905-15	ON063643	ON063842	—	ON063711
	*Fulvoderma australe*	LWZ 20190809-39b	ON063644	ON063843	ON063771	ON063712
	*Fuscoporia gilva*	LWZ 20190814-19b	ON063648	ON063848	ON063775	ON063717
	*Fuscoporia sinica*	LWZ 20190816-19a	ON063649	ON427358	ON063776	ON063719
	*Hydnoporia tabacina*	LWZ 20210924-26a	ON063651	ON063851	ON063778	ON063720
	*Hymenochaete sphaericola*	LWZ 20190808-2b	ON063656	ON063855	ON063783	ON063725
	*Hymenochaete xerantica*	LWZ 20190814-13b	ON063657	ON063856	ON063784	ON063726
	*Inonotus hispidus*	LWZ 20180703-1	ON063659	ON063858	ON063785	ON063727
	*Phellinus piceicola*	LWZ 20190921-5	ON063662	ON063862	ON063790	ON063731
	*Phylloporia oreophila*	LWZ 20190811-27a	ON063665	ON063865	ON063793	ON063733
	*Porodaedalea laricis*	LWZ 20190724-9	ON063668	ON063868	ON063796	ON063735
	*Sanghuangporus weigelae*	LWZ 20210623-2a	ON063671	ON063870	ON063799	ON063736
— / Hyphodontiaceae	*Hyphodontia pachyspora*	LWZ 20170908-5	MT319426	MT319160	—	MT326431
	*Hyphodontia zhixiangii*	LWZ 20170818-13	MT319420	MT319151	—	MT326424
	*Hyphodontia* sp.	LWZ 20170814-15	MT319417	MT319148	—	MT326423
— / Odonticiaceae	*Leifia brevispora*	LWZ 20170820-48	MK343470	MK343474	ON427367	ON463759
	*Leifia flabelliradiata*	KG Nilsson 36270	DQ873635	DQ873635	—	—
	*Leifia* sp.	LWZ 20171015-38	ON427471	ON427354	ON427368	ON463760
	*Odonticium romellii*	KHL s. n.	DQ873639	DQ873639	—	—
— / Peniophorellaceae	*Peniophorella albohymenia*	CLZhao 33187	PQ811412	PQ847496	—	—
	*Peniophorella albohymenia*	CLZhao 33257	PQ811413	—	—	—
	*Peniophorella aspersa*	TNM F24809	MN062097	MN062142	—	—
	*Peniophorella aspersa*	TNM F32708	MN062099	MN062144	—	—
	*Peniophorella cremea*	CLZhao 1606	MT955162	—	—	—
	*Peniophorella cremea*	CLZhao 1719	MT955163	—	—	—
	*Peniophorella crystallifera*	LWZ 20210626-4a	ON063685	ON063885	—	—
	*Peniophorella crystallifera*	TNM F30331	MN062100	MN062147	—	—
	*Peniophorella daweishanensis*	CLZhao 18600	OR094501	OR449932	—	—
	*Peniophorella echinocystis*	KHL 6284	DQ677494	DQ681200	—	—
	*Peniophorella fissurata*	CLZhao 5848	MN864262	OM985777	—	—
	*Peniophorella fissurata*	CLZhao 9421	MN864260	OM985776	—	—
	*Peniophorella guttulifera*	CBS 107303	LT603016	LT603001	—	—
— / Peniophorellaceae	*Peniophorella guttulifera*	NH 12012 (GB)	DQ647501	—	—	—
	*Peniophorella odontiiformis*	TMI 21347	DQ647496	—	—	—
	*Peniophorella odontiiformis*	TMI 6824	DQ647500	—	—	—
	*Peniophorella olivacea*	CLZhao 25896	OR094502	OR449933	—	—
	*Peniophorella pallida*	UC 2022844	KP814208	—	—	—
	*Peniophorella pallida*	UC 2022887	KP814201	—	—	—
	*Peniophorella pertenuis*	NH 12429 (GB)	DQ647486	—	—	—
	*Peniophorella pertenuis*	NH 15115 (GB)	DQ647487	—	—	—
	*Peniophorella praetermissa*	NH 10986 (GB)	DQ647462	—	—	—
	*Peniophorella praetermissa*	NH 11192 (GB)	DQ647461	—	—	—
	*Peniophorella praetermissa*	LWZ 20180903-14	ON063686	ON063886	ON063816	ON063749
	*Peniophorella pubera*	CBS:464.86	MH861988	MH873680	—	—
	*Peniophorella pubera*	LWZ 20210624-16b	ON063687	ON063887	ON063817	ON063750
	*Peniophorella punctata*	CLZhao 33720	PQ811414	PQ847497	—	—
	*Peniophorella punctata*	CLZhao 33732 *	PQ811415	PQ847498	—	—
	*Peniophorella reticulate*	CLZhao 17066	OM985746	OM985783	—	—
	*Peniophorella reticulate*	TNM F22559	MN062103	MN062151	—	—
	*Peniophorella rude*	LWZ 20171026-7	ON063688	ON063888	ON063818	ON063751
	*Peniophorella subpraetermissa*	LWZ 20190816-3b	ON063689	ON063889	ON063819	ON063752
	*Peniophorella subpraetermissa*	Wu 950627	DQ647493	—	—	—
	***Peniophorella subalbohymenia***	**QYZhang 141**	**PX270289**	**PX270293**	—	—
	***Peniophorella subalbohymenia***	**QYZhang 191**	**PX270290**	**PX270294**	—	—
	*Peniophorella yunnanensis*	CLZhao 4810	MN864263	OM985788	—	—
	*Peniophorella yunnanensis*	CLZhao 6137	MN864266	—	—	—
— / Repetobasidiaceae	*Repetobasidium conicum*	KHL 12338	DQ873647	DQ873647	DQ873646	—
	*Repetobasidium mirificum*	FP-133558-sp	—	AY293208	AY293155	AY293243
— / Resiniciaceae	*Resinicium austroasianum*	LWZ 20191208-11	ON063691	ON063891	ON063821	ON063753
	*Resinicium bicolor*	AFTOL-810	DQ218310	—	—	—
	*Resinicium friabile*	LWZ 20210923-23a	ON063692	ON427362	ON063822	ON063754
— / Rickenellaceae	*Rickenella danxiashanensis*	GDGM45513	MF326424	—	ON063823	ON063755
	*Rickenella fibula*	PBM 2503 (AFTOL 486)	DQ241782	MF318953	MF319021	—
— / Rigidoporaceae	*Bridgeoporus sinensis*	Cui 10013	KY131832	KY131891	—	—
	*Leucophellinus hobsonii*	Cui 6468	KT203288	KT203309	—	KT203330
	*Leucophellinus irpicoides*	Yuan 2690	KT203289	KT203310	—	KT203331
	*Rigidoporus cirratus*	LWZ 20170818-16	ON427472	ON427355	ON427369	ON463761
	*Rigidoporus populinus*	LWZ 20190811-39a	ON063674	ON063874	ON063803	ON063740
	*Rigidoporus* sp.	LWZ 20170815-52	ON427473	ON427356	ON427370	ON463762
— / Schizocorticiaceae	*Schizocorticium lenis*	LWZ 20180921-7	ON063827	ON063696	ON063896	ON063760
	*Schizocorticium lenis*	LWZ 20180922-39	MW414525	MW414471	ON427374	ON463764
	*Schizocorticium magnosporum*	Wu 1510-34	MK405351	MK405337	—	—
	*Schizocorticium mediosporum*	Chen 2456	MK405359	MK405345	—	—
	*Schizocorticium parvisporum*	GC 1508-127	MK405361	MK405347	—	—
— / Schizoporaceae	*Fasciodontia brasiliensis*	MSK-F 7245a	MK575201	MK598734	—	—
	*Fasciodontia yunnanensis*	LWZ 20190811-50a	ON063675	ON427360	ON063804	ON063741
	*Fasciodontia* sp.	LWZ 20201011-37	ON063676	ON427361	ON063805	ON063742
	*Lyomyces crustosus*	LWZ 20170815-23	MT319465	MT319201	—	MT326446
— / Schizoporaceae	*Lyomyces leptocystidiatus*	LWZ 20170814-14	MT319429	MT319163	—	MT326512
	*Lyomyces sambuci*	LWZ 20180905-1	MT319444	MT319178	ON063807	MT326438
	*Lyomyces* sp.	LWZ 20180906-20	ON063678	ON063878	ON063808	ON063743
	*Xylodon nesporii*	LWZ 20190814-17a	ON063679	ON063879	ON063809	—
	*Xylodon ovisporus*	LWZ 20190817-6b	ON063680	ON063880	ON063810	ON063744
	*Xylodon rimosissimus*	LWZ 20180904-28	ON063682	ON063882	ON063812	ON063745
	*Xylodon serpentiformis*	LWZ 20190816-12a	ON063683	ON063883	ON063813	ON063746
— / Sideraceae	*Sidera minutipora*	Cui 16720	MN621349	MN621348	MW418078	MW424986
	*Sidera srilankensis*	Dai 19654	MN621344	MN621346	MW418087	MW424989
	*Sidera tenuis*	Dai 18697	MK331865	MK331867	MW418083	MW424988
	*Sidera vulgaris*	Dai 21057	MW198484	MW192009	MW418090	MW424987
— / Skvortzoviaceae	*Skvortzovia dabieshanensis*	LWZ 20210918-15b	ON063694	ON063894	ON063825	ON063757
	*Skvortzovia pinicola*	LWZ 20210623-18b	ON063695	ON063895	ON063826	ON063758
	*Skvortzovia qilianensis*	LWZ 20180904-20	ON063693	ON063893	ON063824	ON063756
	*Skvortzovia yunnanensis*	CLZhao 16084	MW472754	MW473473	—	ON063759
— / Tubulicrinaceae	*Tubulicrinis calothrix*	LWZ 20210919-1b	ON063704	ON063904	ON063835	ON063766
	*Tubulicrinis glebulosus*	LWZ 20180903-13	ON063705	ON063905	ON063836	—
	*Tubulicrinis subulatus*	LWZ 20190914-7	ON063706	ON063906	ON063837	ON063767
—/Trichaptaceae	*Trichaptum biforme*	LWZ 20210919-32a	ON063701	ON063901	ON063832	ON063764
	*Trichaptum fuscoviolaceum*	LWZ 20210918-5b	ON063703	ON063903	ON063834	ON063765
	*Trichaptum perrottetii*	JV 1808/ 101	OQ449091	OQ449030	OQ449404	OQ517072
	*Trichaptum perrottetii*	JV 1908/ 45	OQ449092	OQ449031	OQ449405	OQ874776
	*Trichaptum perrottetii*	B2626	OQ449093	OQ449032	OQ449406	—
—/Umbellaceae	*Umbellus sinensis*	LWZ 20190615-27	OR242616	OR236212	OR240268	OR250300
	*Umbellus sinensis*	LWZ 20190615-39	OR242617	OR236213	OR240269	—
— / Incertae sedis	*Alloclavaria purpurea*	H:6047663	MF319055	MF318905	MF318995	—
	*Alloclavaria purpurea*	M. Korhonen 10305	MF319044	MF318895	MF318986	—
	*Atheloderma mirabile*	TAA 169235	DQ873592	DQ873592	—	—
	*Blasiphalia pseudogrisella*	P. Joijer 4118	MF319047	MF318898	MF318989	—
	*Bryopistillaria sagittiformis*	IO.14.164	MT232349	MT232303	—	—
	*Cantharellopsis prescotii*	H6059300	MF319051	MF318903	MF318993	—
	*Contumyces rosellus*	MGW 1462	MF319059	MF318912	MF319001	—
	*Contumyces vesuvianus*	203608	—	MF318913	MF319002	—
	Cotylidia aurantiaca var. alba	RV.PR98/28	—	AF261458	—	—
	*Cotylidia aurantiaca*	MCR.33	—	AF261460	—	—
	*Cotylidia carpatica*	TENN:071486	MF319060	MF318914	—	—
	*Cotylidia pannosa*	MJ05—1005	JN649334	JN649334	—	—
	*Cotylidia undulata*	H:6059287	MF319064	MF318920	—	—
	*Cotylidia undulata*	H:6059288	MF319061	MF318916	MF319000	—
	*Cotylidia undulata*	IO.15.126	MT232350	MT232304	—	—
	*Cotylidia undulata*	TENN:071491	MF319062	MF318917	—	—
	***Spongoides fissurata***	**QYZhang 190**	**PX270291**	**PX270295**	**PX271056**	**PX275524**
	***Spongoides fissurata***	**QYZhang 194**	**PX270292**	**PX270296**	**PX271057**	**PX275524**
	*Ginnsia viticola*	Wu 0010-29	MN123802	GQ470670	—	—
— / Incertae sedis	*Globulicium hiemale*	Hjm 19007	DQ873595	DQ873595	—	—
	*Globulicium hiemale*	KHL 961221	EU118626	EU118626	—	—
	*Gyroflexus brevibasidiata*	IO.14.230	MT232351	MT232305	—	—
	*Hastodontia halonata*	HHB-17058	MK575207	MK598738	—	—
	*Hastodontia hastata*	KHL 14646	MH638232	MH638232	—	—
	*Lawrynomyces capitatus*	KHL 8464	DQ677491	DQ677491	—	—
	*Loreleia marchantiae*	Lutzoni 930826-1	U66432	U66432	—	—
	*Lyoathelia laxa*	Spirin 8810a	MT305998	MT305998	—	—
	*Muscinupta laevis*	V. Haikonen 19745	MF319066	MF318921	MF319004	—
	*Neocotylidia bambusicola*	Li160910-01 (Type)	OQ376551	OQ372914	—	—
	*Neocotylidia bambusicola*	HCL 2021-8-21	OL351627	OL336498	—	—
	*Neocotylidia bambusicola*	YBCNX2021009	OQ376552	OQ372915	—	—
	*Neocotylidia bambusicola*	YBCNX2021028	OQ376553	—	—	—
	*Neocotylidia diaphana*	DAOM182136	—	AF261459	—	—
	*Neocotylidia diaphana*	TENN:071490	—	MF318915	—	—
	*Neocotylidia fibrae*	AFTOL-700	AY854079	AY629317	AY705958	FJ436111
	*Neocotylidia fibrae*	BJTC FM639 (Type)	MW485002	MW485000	—	—
	*Neocotylidia fibrae*	Li20220715-05	PP762038	PP762043	—	—
	*Neocotylidia fibrae*	Li20220715-08	PP762039	PP762041	—	—
	*Neocotylidia fibrae*	Li20220715-10	PP762040	PP762042	—	—
	*Sphaerobasidium minutum*	KHL 11714	DQ873652	DQ873653	—	—
	*Tsugacorticium kenaicum*	CFMR HHB17347	—	JN368221	JN368234	JN368203
**Polyporales** / Fomitopsidaceae	*Fomitopsis pinicola*	AFTOL 770	AY854083	AY684164	AY705967	FJ436112
— / Grifolaceae	*Grifola frondosa*	AFTOL 701	AY854084	AY629318	AY705960	—
**Thelephorales** / Bankeraceae	*Boletopsis leucomelaena*	PBM2678	DQ484064	DQ154112	DQ435797	—
— / Thelephoraceae	*Thelephora ganbajun*	ZRL20151295	LT716082	KY418908	KY418962	—

Maximum Likelihood (ML) and Bayesian Inference (BI) methods were used for the phylogenetic analyses. The ML analysis was carried out with RAxML version 8.2.12 (Stamatakis 2014), which statistical support values were obtained by using rapid bootstrapping with 1000 replicates, with default settings for other parameters and the best-fit models. The BI tree reconstruction was carried out with MrBayes v. 3.2.5 (Ronquist and Huelsenbeck 2003), which the best-fit partitioning scheme and substitution model were determined by using ModelFinder (Kalyaanamoorthy et al. 2017; Zhang et al. 2020) via the “greedy” algorithm, and branch lengths were estimated as “linked” and AICc. Four Markov chains were run for two runs from random starting trees for 10 million generations and trees were sampled every 1000 generations. The burn-in was set to discard 25% of the trees. A majority rule consensus tree of all remaining trees was calculated. Branches that received bootstrap support for Maximum Likelihood (ML) and Bayesian Posterior Probabilities (BPP) greater than or equal to 75% (ML) and 0.95 (BPP) were considered as significantly supported.

## ﻿Results

### ﻿Phylogeny

**Figure 1.**
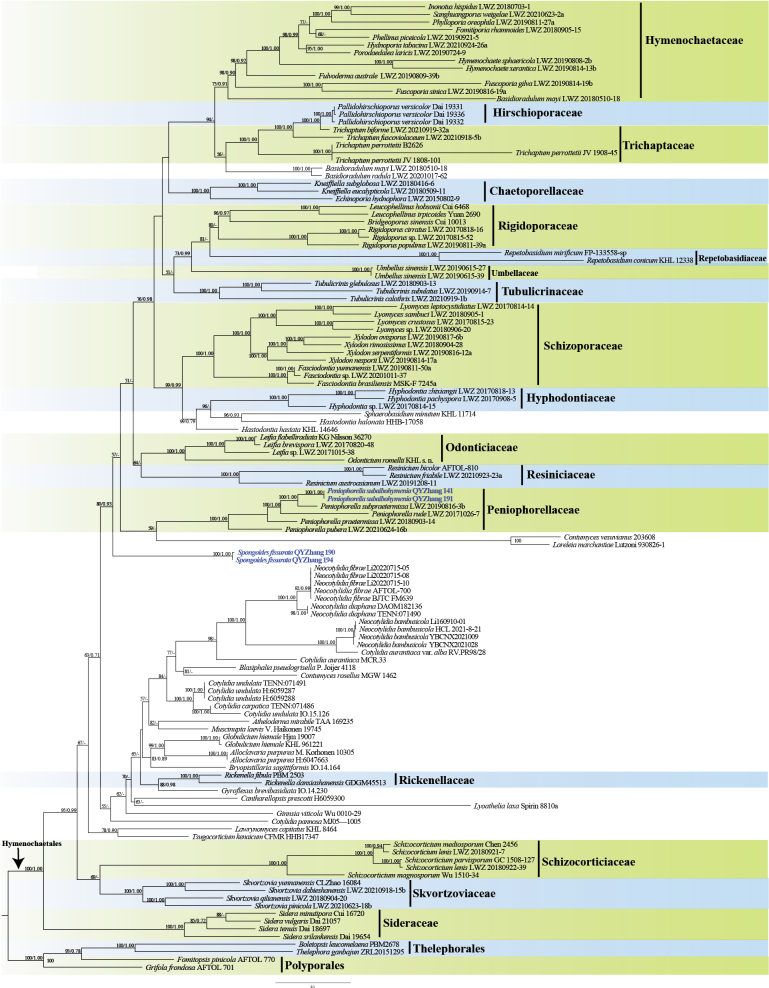
Maximum Likelihood (ML) tree illustrating the phylogeny of the order Hymenochaetales, based on a combined ITS + nrLSU + nrSSU + mtSSU dataset. Branches are labelled with parsimony bootstrap values (ML) higher than 50% and Bayesian Posterior Probabilities (BPPs) more than 0.70.

In this study, the combined ITS + nrLSU + nrSSU + mtSSU dataset (Fig. 1) included sequences from 118 specimens, representing 103 species of Hymenochaetales and *Boletopsis leucomelaena* (Pers.) Fayod and *Thelephora ganbajun* M. Zang from the order Thelephorales, and *Fomitopsis pinicola* (Sw.) P. Karst. and *Grifola frondosa* (Dicks.) Gray from order Polyporales as the outgroups. The dataset had an aligned length of 4,102 characters including gaps, consisting of 1,165 characters from ITS, 927 characters from nrLSU, 1,101 characters from nrSSU, and 909 characters from mtSSU (Table 1). ModelFinder suggested models were GTR + F + I + G4 for ITS, nrLSU and nrSSU, GTR + F + G4 for mtSSU, for the Bayesian analysis. The BI analysis resulted in a concordant topology with an average standard deviation of split frequencies of 0.009808. The ML and BI analyses resulted in nearly identical topologies and only the ML tree is presented with the bootstrap supports for ML and BPP not less than 50% and 0.70, respectively. In Fig. 1, our analysis recognizes 15 families and some genera have no definite position at the family level were recognized in the Hymenochaetales, confirming the results presented by Wang and Zhou (2023) and Ma et al. (2025). The two undescribed specimens (QYZhang 190 and QYZhang 194) formed a distinct, well-supported lineage, phylogenetically distant from known Hymenochaetales families and positioned within incertae sedis. This lineage close to Odonticiaceae L.W. Zhou & X.Wei Wang and Repetobasidiaceae Jülich, but it is not stable and has a low support values (54/-). In addition, two undescribed specimens (QYZhang 141 and QYZhang 191) consistently clustered within the *Peniophorella* clade, forming a highly supported lineage (100/1.00).

**Figure 2.**
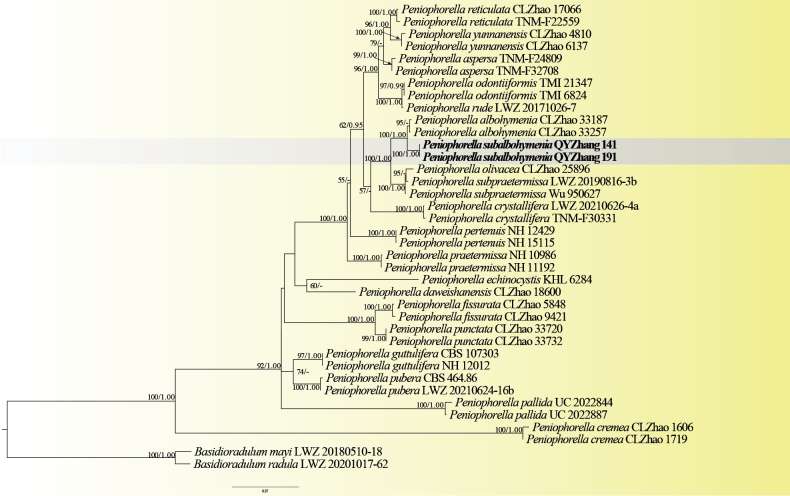
Maximum Likelihood (ML) tree illustrating the phylogeny of *Peniophorella*, based on a combined ITS + nrLSU dataset. Branches are labelled with parsimony bootstrap values (ML) higher than 50% and Bayesian Posterior Probabilities (BPPs) more than 0.70.

The combined ITS + nrLSU dataset (Fig. 2) included sequences from 38 specimens, representing 20 species of *Peniophorella* and two species of *Basidioradulum mayi* X.Wei Wang & L.W. Zhouand and *B. radula* (Fr.) Nobles as the outgroups. The dataset had an aligned length of 1,517 characters, including 655 characters from ITS and 862 characters from nrLSU (Table 1). For the Bayesian analysis, ModelFinder suggested GTR + F + I + G4 as the optimal substitution model for both ITS and nrLSU. The BI analysis resulted in a concordant topology with an average standard deviation of split frequencies of 0.003108. The ML and BI analyses resulted in nearly identical topologies and only the ML tree is presented with the bootstrap supports for ML and BPP not less than 50% and 0.70, respectively. In Fig. 2, the phylogram inferred from ITS + nrLSU sequences within *Peniophorella* (Fig. 2) highlighted two undescribed specimens (QYZhang 141 and QYZhang 191) formed an independent lineage with a robust support (100/1.00), and closely related to *P. albohymenia* Y.L. Deng & C.L. Zhao.

### ﻿Taxonomy

#### ﻿ Spongoides

Taxon classificationFungiHymenochaetalesRickenellaceae

Q.Y. Zhang gen. nov.

BD736A62-D064-50EA-A50C-9EBC1440D42F

861199

##### Etymology.

Spongoides (Lat.): derived from the Latin spongos (sponge) and the suffix-eides (resembling), referring to the spongy texture of the basidiomata.

##### Type species.

*Spongoides fissurata* Q.Y. Zhang, sp. nov.

##### Description.

Basidiomata annual, resupinate, effused, closely adnate, inseparable from substrate, thick, spongy. Hymenophore smooth, with wrinkles or cracks, white to cream; margin thinning out or abrupt, adnate. Hyphal system monomitic; generative hyphae clamped, colorless, thin walled, frequently branched, septate. Hymenium two kinds of cystidia, tapering or bottled, colorless, thin-walled. Basidia cylindrical, with a basal clamp connection and four sterigmata, filled with refractive oil-like matter. Basidiospores cylindrical or ellipsoid with an apiculus, colorless, thin-walled, smooth, IKI–, CB–, with oily contents.

##### Notes.

Building upon the well-established taxonomic framework of Hymenochaetales (Liu et al. 2024; Ma et al. 2025), our phylogeny confirms that *Spongoides* belongs to the order but is distinct from all known families and genera (Fig. 1). The family position of this genus needs to be further clarified. *Spongoides* seems to be morphologically related to members of Schizocorticiaceae L.W. Zhou & Xue W. Wang; however, *Spongoides* is distinct by the spongy basidiomata and the absence of tubular with obtuse apex leptocystidia. Given the phylogenetic distance and absence of key morphological characteristics possessed by the other genera in Hymenochaetales, we propose the establishment of *Spongoides* as a novel genus within incertae sedis of Hymenochaetales, with *Spongoides fissurata* as its generic type.

#### ﻿ Spongoides fissurata

Taxon classificationFungiHymenochaetalesRickenellaceae

Q.Y. Zhang sp. nov.

153D0601-0696-56DB-9D09-99F4BB708F3D

861200

Figs 3, 4

##### Etymology.

*fissurata* (Lat.): Refers to the cracking hymenial surface of the type specimens.

##### Holotype.

China • Fujian Province, Nanping, Laizhou Town, Lai Zhou Forestry Experiment Station, on living *Chamaecyparis formosensis*, leg. Q.Y. Zhang, 20 June 2025, QYZhang 190 (NJFC).

##### Description.

Basidiomata annual, resupinate, effused, closely adnate, inseparable from substrate, thick, spongy when fresh and dry, without odor or taste when fresh, up to 8 cm long, 4 cm wide, 0.5 cm thick, and with extremely thin layer. Hymenial surface smooth, with wrinkles or cracks, white to light gray when fresh and dry. margin thinning out or abrupt, adnate. Hyphal system monomitic; generative hyphae with clamp connections, thin-walled, colorless, occasionally branched, 2.5–4 μm in diameter, IKI–, CB–, tissues unchanged in KOH. Cystidia of two types: (1) tapering cystidia, thin-walled, 30–49 × 2–4 μm; (2) bottled cystidia, with a relatively long neck, smooth, thin-walled, 23–35 × 3–7 μm. Basidia clavate, with four sterig-mata and a basal clamp connection, colorless, thin-walled, 16–25 × 6–8 μm; basidioles in shape similar to basidia, but slightly smaller. **Basidiospores** ellipsoid, colorless, thin-walled, IKI–, CB–, (6.5–)7–10(–11) × 3–6.4(–7) μm, L = 8.16 μm, W = 4.05 μm, Q = 1.96–2.05 (n = 60/2).

**Figure 3.**
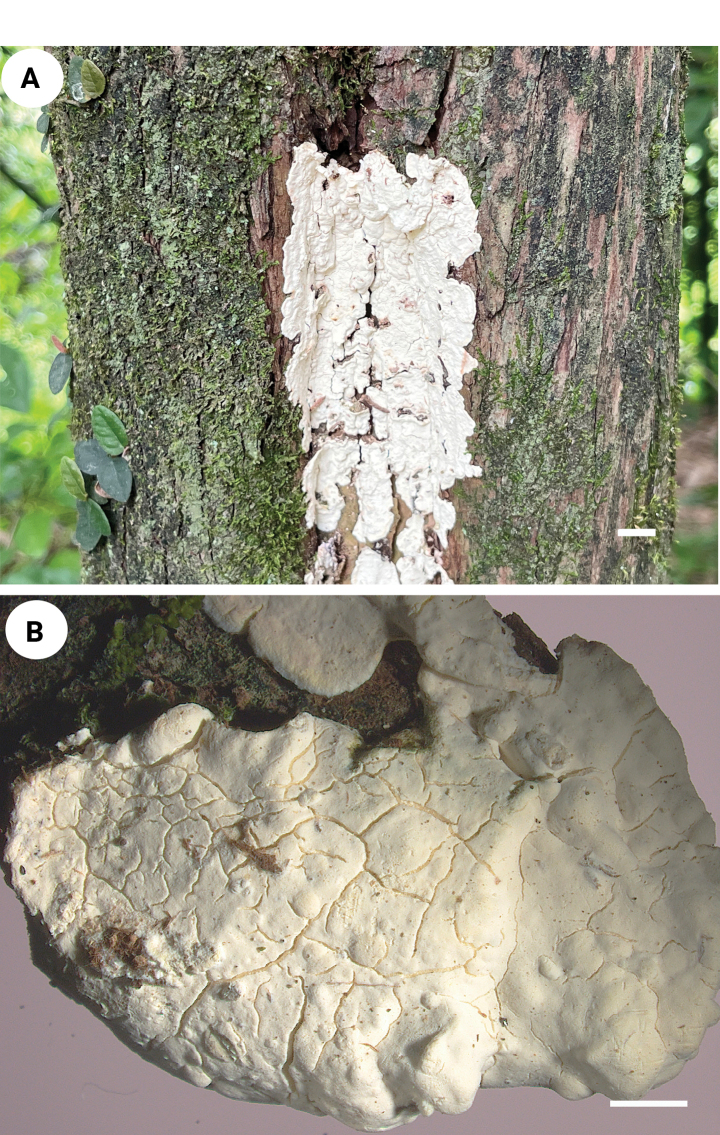
Basidiomata of *Spongoides fissurata* (holotype, QY Zhang 190). Scale bars: 1 cm (**A**); 2 mm (**B**).

##### Additional specimen (paratype) examined.

China • Fujian Province, Nanping, Laizhou Town, Lai Zhou Forestry Experiment Station, on living *Chamaecyparis formosensis*, leg. Q.Y. Zhang, 20 June 2025, QYZhang 194 (NJFC).

**Figure 4.**
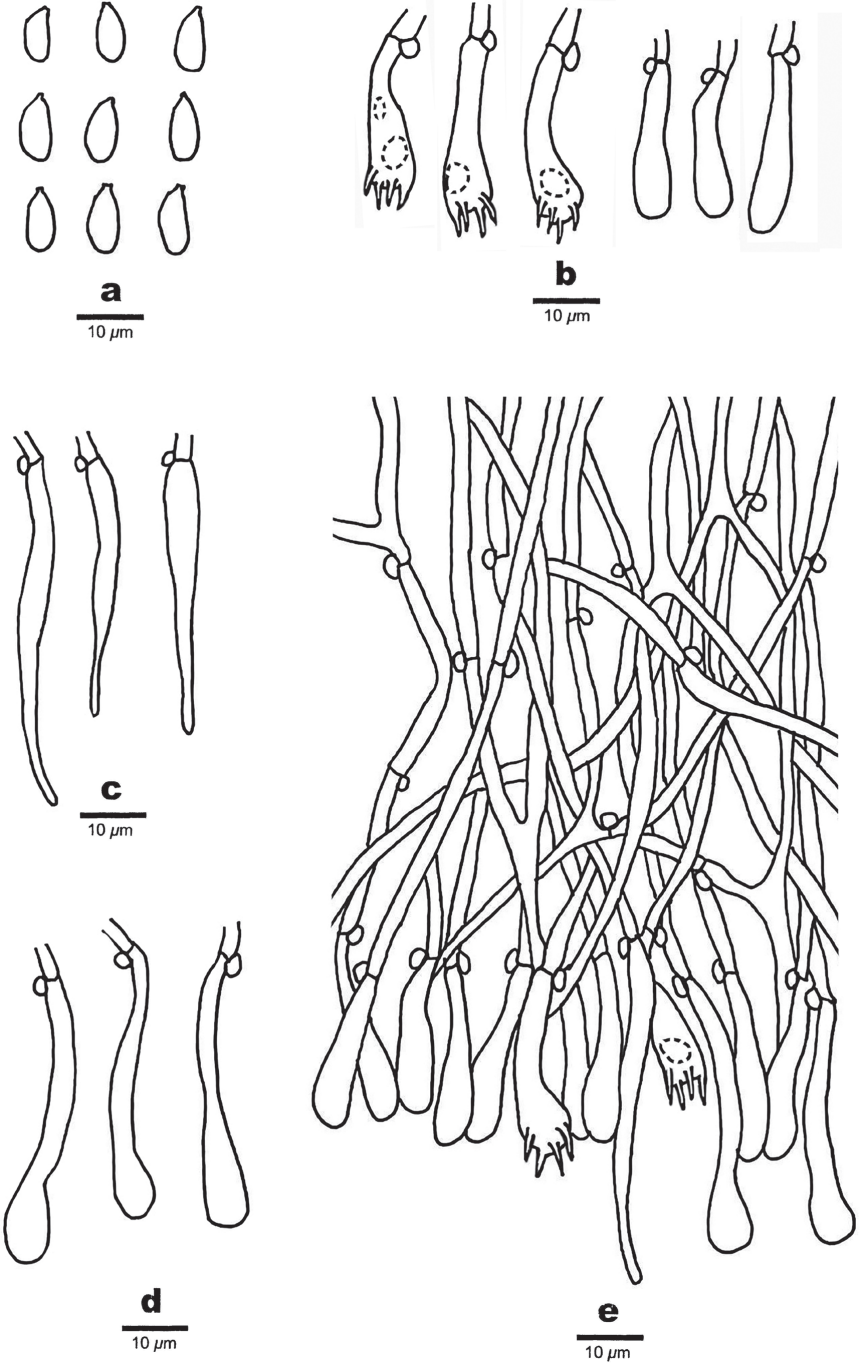
Microscopic structures of *Spongoides fissurata* (holotype, QY Zhang 190). **a.** Basidiospores; **b.** Basidia and basidioles; **c.** Tapering cystidia; **d.** Bottled cystidia; **e.** A section of hymenium.

#### ﻿ Peniophorella subalbohymenia

Taxon classificationFungiHymenochaetalesRickenellaceae

Q.Y. Zhang sp. nov.

798F176C-FCD5-5A39-A6AF-3C357912D715

861201

Figs 5, 6

##### Holotype.

China • Fujian Province, Nanping, Laizhou Town, Lai Zhou Forestry Experiment Station, on fallen angiosperm branch, leg. Q.Y. Zhang, 20 June 2025, QY Zhang 141 (NJFC).

##### Etymology.

*Subalbohymenia* (Lat.): Refers to the morphological similarity and close phylogenetic relationship with *P. albohymenia*.

##### Diagnosis.

*Peniophorella subalbohymenia* is characterized by the membrana-ceous basidiomata with white hymenial surface, three types cystidia as stephanocyst, fusiform cystidia and lageniform cystidia, and ellipsoid basidiospores measuring 5–8 × 3.8–4.5 μm.

**Figure 5.**
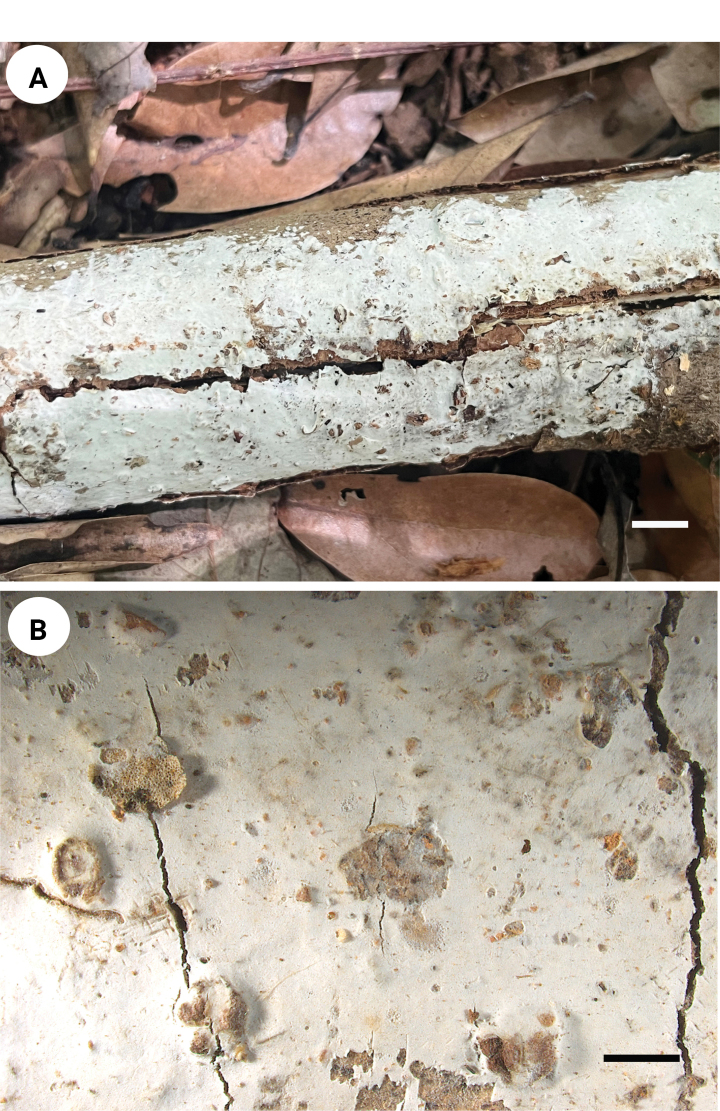
Basidiomata of *Peniophorella subalbohymenia* (holotype, QY Zhang 141). Scale bars: 1 cm (**A**); 2 mm (**B**).

##### Description.

**Basidiomata** annual, resupinate, adnate, membranaceous, with-out odor or taste when fresh, up to 12 cm long, 5 cm wide, and with extremely thin layer. Hymenial surface smooth, white when fresh and dry. Sterile margin distinctly, thin, white, up to 5 mm long. Hyphal system monomitic; generative hyphae with clamp connections, thin-walled, colorless, occasionally branched, 2.5–5 μm in diameter, IKI–, CB–, tissues unchanged in KOH. **Cystidia** of four types: (1) stephanocyst, thin-walled, with a ring-like wart protrusion on the upper-middle part, 13–17 × 9–10 μm; (2) fusiform cystidia, the apical part encrusted with asteroid, smooth, thin-walled, 25–55 × 6–15 μm; (3) lageniform cystidia, thin-walled, with a relatively long neck, 30–60 × 8–10 μm. **Basidia** clavate, with four sterig-mata and a basal clamp connection, colorless, thin-walled, 18–25 × 6.5–8 μm; basidioles in shape similar to basidia, but slightly smaller. **Basidiospores** ellipsoid, colorless, thin-walled, IKI–, CB–, (4.8–)5–8 × (3.5–)3.8–4.5(–4.8) μm, L = 6.14 μm, W = 4.12 μm, Q = 1.45–1.53 (n = 60/2).

**Figure 6.**
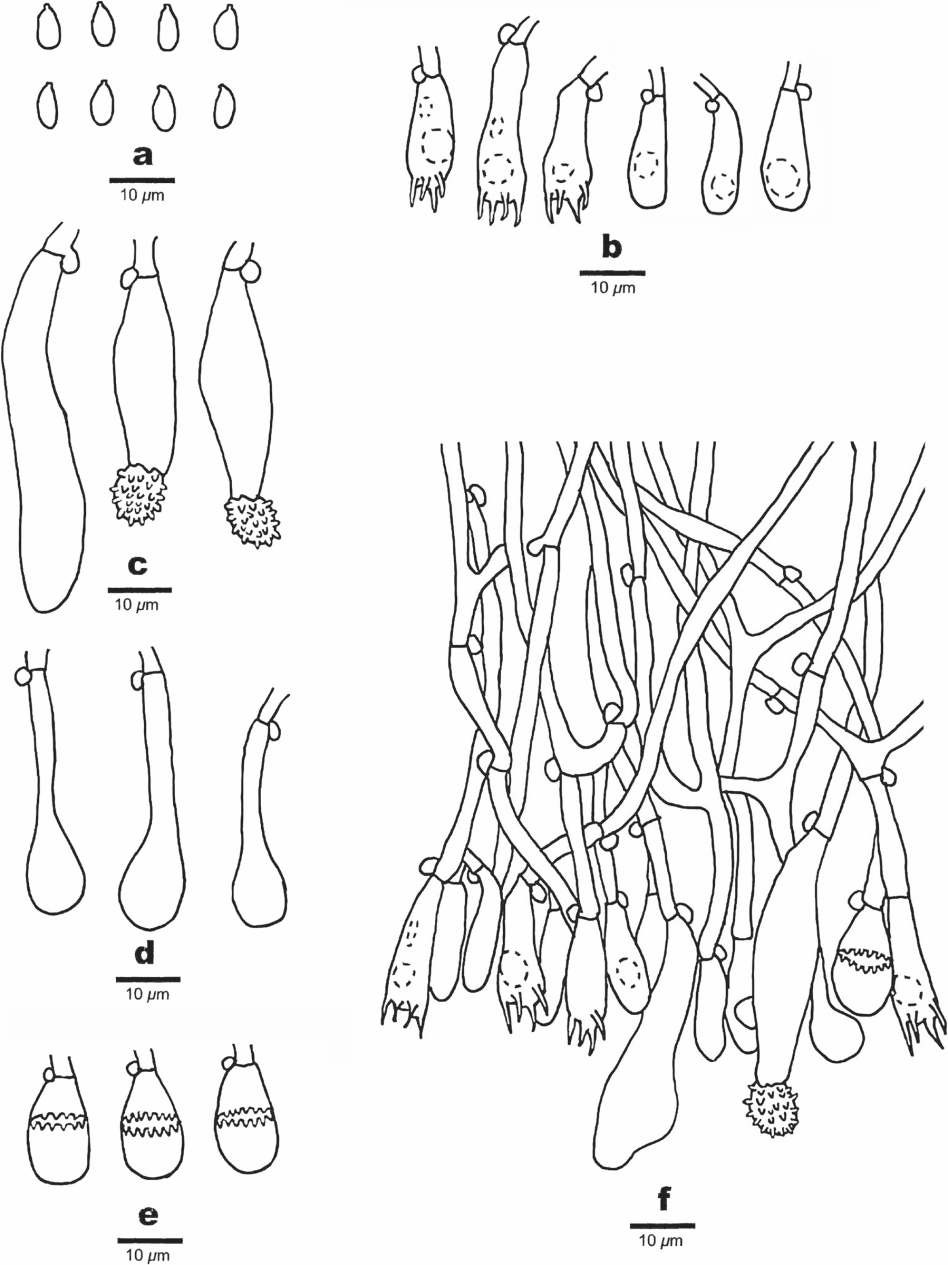
Microscopic structures of *Peniophorella subalbohymenia* (holotype, QY Zhang 141). **a.** Basidiospores; **b.** Basidia and basidioles; **c.** Fusiform cystidia; **d.** Lageniform cystidia **e.** Stephanocyst; **f.** A section of hymenium.

##### Another specimen (paratype) examined.

China • Fujian Province, Nanping, Laizhou Town, Lai Zhou Forestry Experiment Station, on fallen angiosperm branch, leg. Q.Y. Zhang, 20 June 2025, QYZhang 191 (NJFC).

## ﻿Discussion

The order Hymenochaetales represents a well-studied group of wood-inhabiting fungi within the Agaricomycetes (Basidiomycota), characterized by diverse basidiomata including poroid, hydnoid, corticioid, and agaricoid forms. Among these, corticioid fungi represent a significant and morphologically well-defined group within the order. Although the phylogenetic relationships of the order Hymenochaetales have been extensively studied, the results are different depending on the specimens and several gene sequences (Wang et al. 2021, 2023; Wu et al. 2022; Zhou et al. 2023).

At the family level, the classification of Hymenochaetales has been continually emended, especially in the past fifteen years. For example, *Peniophorella* was initially placed within the Rickenellaceae sensu lato based on morphological observation (Larsson 2007; He et al. 2019). Multilocus phylogenetic analyses have since shown that *Peniophorella* constitutes a distinct, well-supported lineage independent from other families in Hymenochaetales, leading to the establishment of the family Peniophorellaceae (Wang et al. 2023). Additionally, Wang et al. (2023) identified 14 families using seven genetic loci. In contrast, Zhou et al. (2023) accepted 11 families based on five genetic loci along with morphological evidence. Meanwhile, Zhao et al. (2025) proposed the recognition of 10 families and the rejection of 2 families based on the phylogenomics analysis. Furthermore, the delimitations of certain families are not fully resolved, and they are therefore regarded as incertae sedis, which encompass the following 19 genera, viz. *Alloclavaria* Dentinger & D.J. McLaughlin, *Atheloderma* Parmasto, *Blasiphalia* Redhead, *Bryopistillaria* Olariaga, Huhtinen, Læssøe, J.H. Petersen & K. Hansen, *Cantharellopsis* Kuype, *Contumyces* Redhead, Moncalvo, Vilgalys & Lutzoni, *Cotylidia* P. Karst., *Ginnsia* Sheng H. Wu & Hallenb., *Globulicium* Hjortstam, *Gyroflexus* Raithelh., *Hastodontia* (Parmasto) Hjortstam & Ryvarden, *Kurtia* Karasiński, *Lawrynomyces* Karasiński, *Loreleia* Redhead, Moncalvo, Vilgalys & Lutzoni, *Lyoathelia* Hjortstam & Ryvarden, *Muscinupta* Redhead, Lücking & Lawrey, *Sphaerobasidium* Oberw., *Subulicium* Hjortstam & Ryvarden, and *Tsugacorticium* Nakasone & Burds.

Phylogenetically, the phylogram based on the combined ITS + nrLSU + nrSSU + mtSSU sequences (Fig. 1) revealed that the new genus *Spongoides* (represented by its type species *Spongoides fissurata*) forms a distinct monophyletic lineage within Hymenochaetales, with uncertain familial placement (incertae sedis). Although this lineage appears phylogenetically close to Odonticiaceae and Repetobasidiaceae, the relationship remains unstable and is supported by low statistical support values. Morphologically, Odonticiaceae species differs from *Spongoides* by its grandinioid, odontioid to hydnoid basidiomata and cylindrical cystidia, while *Spongoides* has thick, spongy basidiomata and two kinds of cystidia, tapering or bottled; and Repetobasidiaceae species differs from *Spongoides* by its thin, ceraceous basidiomata and generative hyphae with clamp connections (Wang et al. 2023). Additionally, eight genera within the incertae sedis of Hymenochaetales exhibit resupinate, membranaceous or corticioid basidiomata with white to yellowish appearances, making them difficult to distinguish based on macromorphological characteristics. A morphological comparison between the new genus *Spongoides* and the other eight genera is presented in Table 2.

**Table 2.** A morphological comparison between *Spongoides* and similar eight genera in the incertae sedis of Hymenochaetales.

Species name	Hymenial surface	Generative Hyphae	Cystidia	Basidia	Basidiospores	References
*** Atheloderma ***	pellicular, smooth, margin whitish, fimbriate or with thin rhizomorphs	monomitic, clamped, thin-walled	cylindrical, basally widened, with obtuse to subcapitate apex	clavate, often constricted	narrowly ellipsoid, colorless, thin-walled, smooth, IKI–, CB–	Eriksson and Ryvarden 1973
*** Ginnsia ***	Pellicular, membranaceous, smooth	Monomitic, simple-septate	present	clavate with stalked bases	ellipsoid, thin-walled, guttulate, IKI–, CB–	Wu et al. 2010
*** Globulicium ***	adnate, ceraceous, margin indeterminate	monomitic, clamps, thin-walled, richly branched	absent, paraphysoid hyphae present, some encrusted	clavate to cylindrical, constricted	globose, thin-walled, smooth, IKI–	Hjortstam 1973
*** Lawrynomyces ***	resupinate, effused, adnate, thin, even, margin indeterminate, without rhizomorphs	monomitic, simple-septa, thin to thick-walled	hyphidia sometimes present	suburniform (utriform) to subcylindrical and constricted, more or less pedunculate	broadly ellipsoid to subglobose, slightly thickened walls and distinct apiculus, smooth, c, CB–	Karasiński 2013
*** Lyoathelia ***	resupinate, loosely attached, pellicular or membranous, smooth, with a thin, whitish subiculum and sparse or distinct hyphae	monomitic, clamped, thin-walled or thick-walled, more or less encrusted	capitata, moderately encrusted	thin-walled, relatively large	thin-walled or moderately thick-walled, smooth​	Hjortstam and Ryvarden 2004
*** Sphaerobasidium ***	resupinate, adnate, effused, very thin, smooth, margin indeterminate.	monomitic, clamped, thin-walled	leptocystidia present	subglobose to obconical	thin-walled, smooth, IKI–, CB–	Liu et al. 2022
*** Spongoides ***	resupinate, effused, closely adnate, thick, spongy, smooth, with wrinkles or cracks	monomitic, clamped, colorless, thin-walled	two kinds of cystidia, 1) tapering; 2) bottled, thin-walled	cylindrical	cylindrical or ellipsoid with an apiculus, colorless, thin-walled, smooth, IKI–, CB–	In present study
*** Subulicium ***	resupinate, effused, adnate, smooth, pilose by projecting cystidia	monomitic, simple-septa, distinct and with thin- to thickened walls	two kinds of cystidia, 1) subulate, lateral, smooth or encrusted, thick-walled; 2) gloeocystidia present or absent,	clavate or subcylindrical	globose to subglobose, with thin- to slightly thickened walls, IKI–, CB–	Hjortstam, and Ryvarden 1979
*** Tsugacorticium ***	effuse, adnate, soft, smooth, with distinct, abrupt margin	monomitic, clamped, smooth, subhymenium thickening, with dendrohyphidia	present	suburniform, elongate	globose to subglobose, thin-walled, smooth, IKI–, CB–	Nakasone and Burdsall 2012

Furthermore, the corticioid fungi represents a highly polyphyletic status, they are not confined to the order Hymenochaetales but are prevalent in several other major orders of Agaricomycetes, such as Polyporales and Russulales. Similarly, some other genera, such as *Hyphoderma* Wallr (Polyporales) and *Peniophora* Cooke (Russulales) may share superficial morphological similarities with *Spongoides*. *Hyphoderma niveomarginatum* Y. Yang & C.L. Zhao is similar to *Spongoides fissurata* by its resupinate, white, cracking hymenial surface and basidiospores of similar size, but it differs from *Spongoides fissurata* by its ceraceous basidiomata and cystidia with contractions of varying degrees (Yang et al. 2023). *Peniophora* is an old corticioid genus comprising a large number of species. Similar to *Spongoides fissurata*, they have pale, resupinate basidiomata, and cause white rot. In contrast, a key distinguishing feature of *Peniophora* is the production of both encrusted cystidia and gloeocystidia (Xu et al. 2023).

In the present study, a new species, *Peniophorella subalbohymenia* is described based on phylogenetic and morphological characters. Phylogenetically, *Peniophorella subalbohymenia* is closely related to *P. albohymenia*. But morphologically *P. subalbohymenia* differs from *P. albohymenia* by having lageniform cystidia measuring 30–60 × 8–10 μm, and smaller basidiospores (5–8 × 3.8–4.5 μm vs. 9–10.9 × 4.5–5 μm, Deng et al. 2025). Also, there are 20 base pairs differences between *Peniophorella subalbohymenia* and *P. albohymenia*, which amounts to > 2% nucleotide differences in the ITS regions. Morphologically, *P. praetermissa*, *P. yunnanensis* C.L. Zhao, are similar to *P. subalbohymenia* based on the smooth hymenophore, allantoid basidiospores, and fusiform cystidia of the apical part encrusted with asteroid. However, *P. praetermissa* differs *P. subalbohymenia* in its longer basidiospores (8–11 μm in length vs. 5–8 μm in length), and the absence of large gloeocystidia measuring 30–120 × 8–18 μm (Hallenberg et al. 2007). *Peniophorella yunnanensis* differs *P. subalbohymenia* in smaller cystidia of the apical part encrusted with asteroid (9–28 × 3–8.5 μm vs. 25–55 × 6–15 μm) and the absence of stephanocyst (Guan et al. 2020).

Macrofungi, particularly wood-rotting fungi, constitute a vital component of forest ecosystems (Wu et al. 2022; Zhao et al. 2024). They produce a wide array of bioactive compounds and enzymes that break down organic matter, facilitating the decomposition of dead plant and animal tissues and enabling nutrient recycling (Wei and Dai 2004; Cui et al. 2018; Hyde 2022; Zhou et al. 2025). As such, they play an essential role in sustaining the biosphere, and play a core role in ecosystem processes and functioning. In recent years, with the rapid development of genome sequencing technologies at more affordable costs, the study of the phylogenetic relationships has attracted widespread attention due to its provision of higher species resolution. Studies on the taxonomy and phylogeny of wood-decomposing fungi have made significant progress. While numerous new species and genera within the order Hymenochaetales have been reported and described (Wu et al. 2022; Zhou et al. 2023; Liu et al. 2025), many novel taxa remain undiscovered, particularly in subtropical and tropical regions. The fungal species diversity in Hymenochaetales still has considerable potential for development. In addition, more undescribed Hymenochaetales records will be discovered throughout China after extensive collection combined with morphological and molecular analyses.

## Supplementary Material

XML Treatment for Spongoides

XML Treatment for Spongoides fissurata

XML Treatment for Peniophorella subalbohymenia

## ﻿Additional information

### Use of AI

No use of AI was reported.

### Author ORCIDs

Qiu-Yue Zhang https://orcid.org/0000-0001-9458-3566

Li-Hua Zhu https://orcid.org/0000-0003-2740-4980

Lin Huang https://orcid.org/0000-0001-7536-0914

### Data availability

The new sequences are deposited in the GenBank database (Table 1).

## Supplementary materials

Supplementary material 1 Bayesian Inference (BI) tree Data type pdf https://binary.pensoft.net/file/1506505This dataset is made available under the Open Database License (http://opendatacommons.org/licenses/odbl/1.0/). The Open Database License (ODbL) is a license agreement intended to allow users to freely share, modify, and use this Dataset while maintaining this same freedom for others, provided that the original source and author(s) are credited.Qiu-Yue Zhang, Jin-Hua Huang, Jian-Ling Ren, Li-Hua Zhu, Lin Huang
