# Supplementary figures and images for: ﻿Morphological and phylogenetic analyses reveal a new genus and two new species of Hymenochaetales (Basidiomycota) from southeast China
Source: MycoKeys. 2026 Jan 6;127:25–46. doi: 10.3897/mycokeys.127.171179 (PMC12800780; doi:10.3897/mycokeys.127.171179)

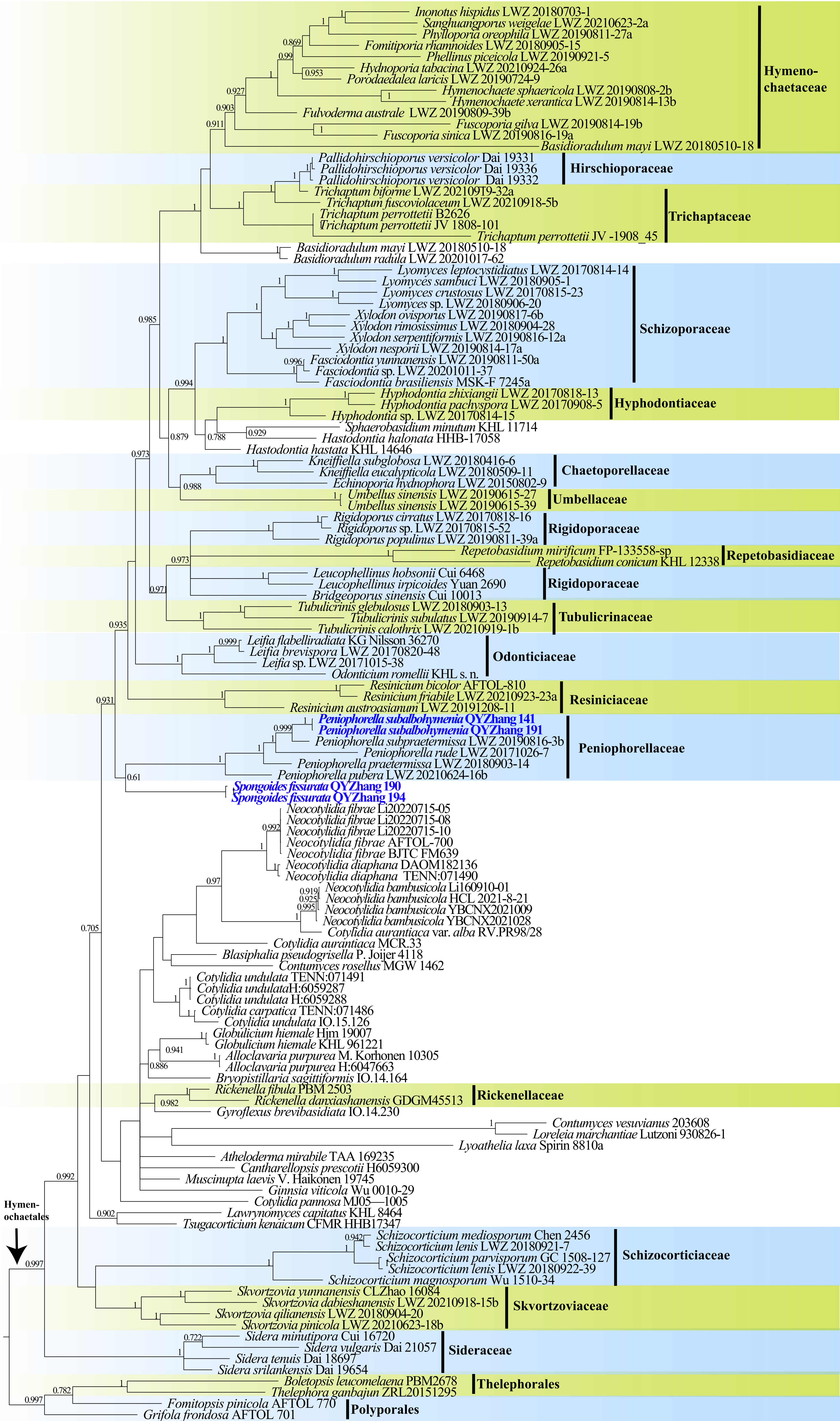

Supplement: Supplementary material 1 — Bayesian Inference (BI) tree [file mycokeys-127-025-s001.pdf]
